# Supplementary material for: The interactome of intact mitochondria by cross-linking mass spectrometry provides evidence for coexisting respiratory supercomplexes
Source: Mol Cell Proteomics. 2017 Dec 8;17(2):216–32. doi: 10.1074/mcp.RA117.000470 (PMC5795388; doi:10.1074/mcp.RA117.000470)
Supplement: Supplemental Data [file supp_RA117.000470_133922_0_supp_23978_fzffwf.zip › spectra_annotation/mito_spectra_annotation/59-1-6-1-17-1.pdf]

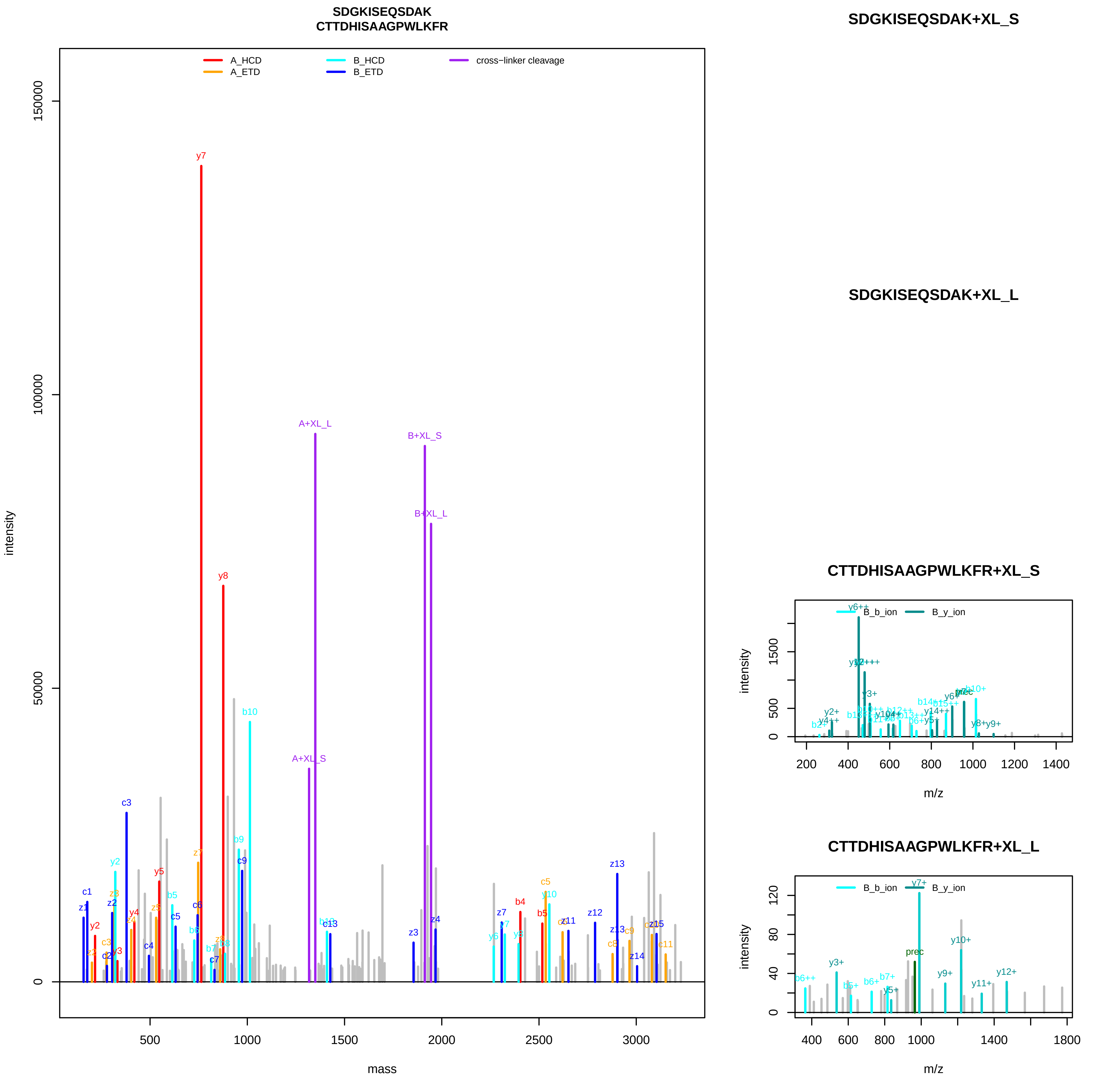
SDGKISEQSDAK
CTTDHISAAGPWLKFR
A_HCD
A_ETD
B_HCD
B_ETD
cross−linker cleavage
intensity
mass
0
50000
100000
150000
500
1000
1500
2000
2500
3000
y7
y8
A+XL_L
B+XL_S
B+XL_L
A+XL_S
b10
b9
c9
c3
y2
z7
c13
z13
c5
y10
b4
b5
z12
z11
z4
z3
c1
z1
z2
y5
c6
b5
c5
y4
z4
y2
c2
y3
c4
b6
c7
b7
z8
b8
b12
y6
y7
y8
z15
c11
c8
c9
z14
SDGKISEQSDAK+XL_S
SDGKISEQSDAK+XL_L
CTTDHISAAGPWLKFR+XL_S
B_b_ion
B_y_ion
intensity
m/z
0
500
1500
200
400
600
800
1000
1200
1400
y6++
y2+
y4++
b2+
y3+
b14++
b15++
y14++
y5+
y6+
prec
b10+
y8+
y9+
CTTDHISAAGPWLKFR+XL_L
B_b_ion
B_y_ion
intensity
m/z
0
40
80
120
400
600
800
1000
1400
1800
y7+
prec
y10+
y3+
b6++
b5+
b6+
b7+
y5+
y9+
y11+
y12+
